# Current diagnostics and treatment of the cubital tunnel syndrome in Austria

**DOI:** 10.3205/iprs000082

**Published:** 2016-01-18

**Authors:** Kristina Harder, Jens Diehm, Isabella Fassola, Nesrin Al khaled, Dietrich Doll, Sebastian E. Dunda, Björn Dirk Krapohl

**Affiliations:** 1 Department of Plastic and Hand Surgery, St. Marien-Krankenhaus Berlin, Germany; 2 Center for Musculoskeletal Surgery, Charité – Medical University of Berlin, Germany

**Keywords:** cubital tunnel syndrome, ulnar nerve, peripheral nerve compression

## Abstract

According to the vote of the Austrian Society for Surgery of the Hand (ÖGH) an investigation to collect data on the current state of the treatment of cubital tunnel syndrome was initiated. Over one year a total of 875 patients with cubital tunnel syndrome were operated in Austria, this means an incidence of this nerve entrapment of 0.011%. Most of the operations were done by trauma surgeons (287; 33%). For diagnosis most of the centers rely on clinical symptoms, electroneurophysiology, and elbow X-ray. 40% of the institutions regard conservative therapy as useless and not indicated. If conservative treatment modalities are applied, physiotherapy (97%), non-steroidal anti-inflammatory medication (77%), and glucocorticoid injections (30%) are primarily used. In case of simple nerve entrapment most of the surgeons (72%) prefer simple nerve decompression. If there is additional pathology subcutaneous cubital nerve transposition is recommended (62%). Endoscopic techniques are only use by 3% of the surgeons. In the postoperative care, physiotherapy is favored in 51%, whereas 24% do not judge any postoperative care as beneficial.

The three most often encountered complications were incomplete remission, scar contracture and hypertrophy, and postoperative bleeding.

## Zusammenfassung

In Absprache mit der Österreichischen Gesellschaft für Handchirurgie (ÖGH) wurde eine Erhebung zum Stand der Diagnostik und Therapie bei Sulcus-nervi-ulnaris-Syndrom in Österreich durchgeführt. Mit der vorliegenden Arbeit wurde das Ergebnis dieser Erhebung ausgewertet. Insgesamt wurden im Jahr 875 Sulcus-nervi-ulnaris-Syndrome operiert, das entspricht einer angenäherten Erkrankungsinzidenz von 0,011%. Die meisten Operationen wurden von Unfallchirurgen durchgeführt (287; 33%). In der Diagnostik werden neben klinischer Symptomatik vorwiegend Elektroneurophysiologie und Ellenbogennativröntgen berücksichtigt. 40% der Befragten halten einen konservativen Therapieversuch für nicht indiziert. Von den konservativen Therapieoptionen haben Physiotherapie (97%), nichtsteroidale Antirheumatika (77%) und Glukokortikoidinjektionen (30%) die Priorität. Bei einfachem Kompressionssyndrom bevorzugt die Mehrzahl (72%) eine einfache Dekompression des Nerven. Bei zusätzlicher Pathologie am Ellenbogen wird der subkutanen Ventralverlagerung der Vorzug gegeben (62%). Endoskopische Techniken werden nur von 3% der Operateure verwendet. In der Nachbehandlung steht die Physiotherapie mit 51% an erster Stelle, 24% erachten jegliches Nachbehandlungssystem für nicht erforderlich.

Die drei wesentlichen Komplikationen waren unvollständige Remission, kontrakte und hypertrophe Narben sowie Nachblutungen.

## Introduction

Ulnar nerve entrapment mostly arises in the elbow region and is the second most commonly seen entrapment neuropathy after carpal tunnel syndrome. This disorder called cubital tunnel syndrome is of multifactorial origin and many treatment methods have been defined. Still unsure is the optimal treatment in particular concerning the kind of surgical intervention. The results of a survey by the Austrian Society of Hand Surgery documents the current situation.

## Material and methods

The Austrian Society for Surgery of the Hand (ÖGH) initiated a global survey in Austria to record the current situation of the treatment of the cubital tunnel syndrome in Austria. A questionnaire with discriminating items was created and sent to all medical institutions in Austria performing surgical treatment on cubital tunnel syndrome including general surgeons, trauma and orthopedic surgeons, plastic surgeons, and neurosurgeons. The aim was to discriminate the most common surgical procedures, detect the differences of the modalities between different faculties and catch the current way of pre- and postoperative diagnostic and therapeutic steps. Four sections were defined:

- A: Institutional parameters concerning case load, generally and concerning cubital tunnel syndrome
- B: Pre-operative diagnostic and conservative treatment performed
- C: Applied surgical techniques, favorite alternatives, and, if performed, endoscopical procedures
- D: Post-operative care, outcome, complications and follow-up

Section A was defined to find out the number of surgeries for cubital tunnel syndrome in common. The different institutions were asked for their number of cubital tunnel syndrome surgeries and the number of all surgeries performed during the period.

Section B concerning the peri-operative care included questions about the usually performed diagnostics (clinical examination, X-ray, electrophysiological testing (EMG, NLG), CT scan, MRI, ultrasound). It also asked about the regular duration of the symptoms and if a conservative treatment was an option.

Section C focused on the way of surgical treatment. Common alternatives are mere decompression of the nerve, additional shift with either subcutaneous, intra- or transmuscular positioning, or epicondylectomy.

**Figure 1:**
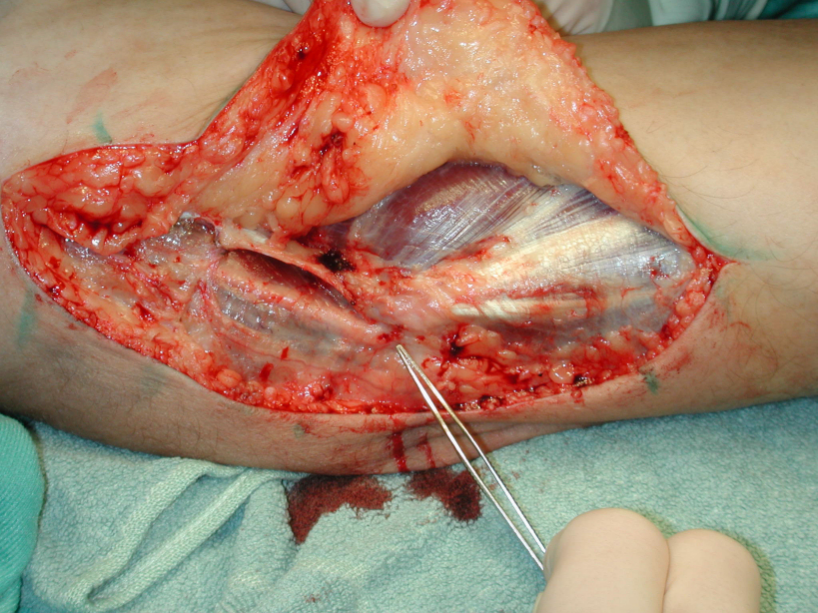
Decompression of the ulnar nerve

**Figure 2:**
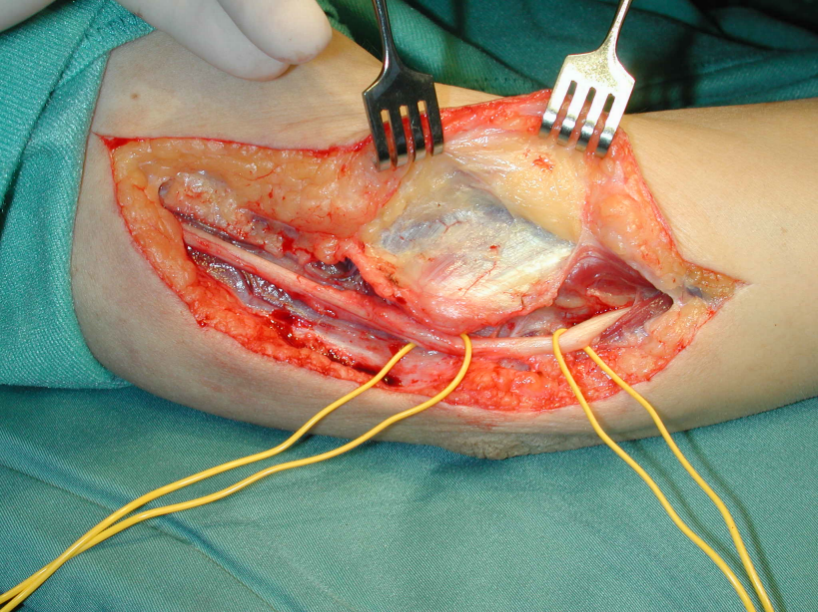
Mobilization of the ulnar nerve over a long distance

**Figure 3:**
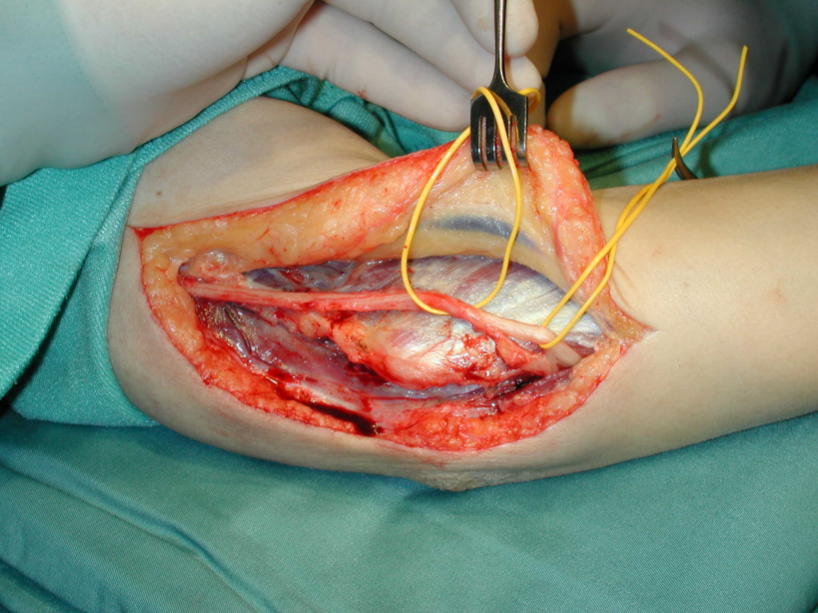
Subcutaneous shift/transposition of the ulnar nerve

Figure 1 (Fig. 1), Figure 2 (Fig. 2) and Figure 3 (Fig. 3) show the intraoperative situs for nerve decompression und subcutaneous nerve transposition.

For post-operative care, we defined parameters: duration and kind of immobilization, medication, and the major complications. And at last the institutions were asked for their follow-up strategy, if it was done regularly by clinical examination or with technical support.

## Results

**Figure 4:**
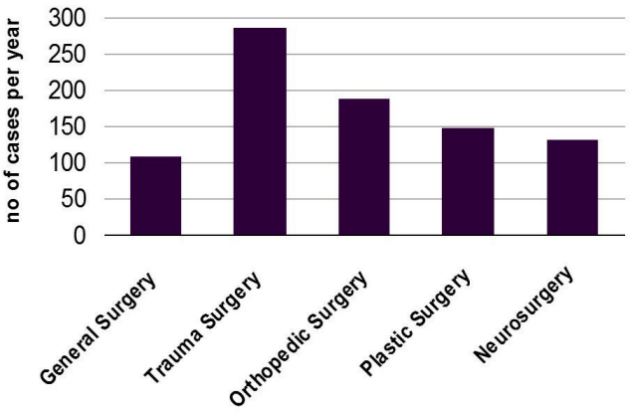
Distribution of the surgical specialties performing operative treatment of cubital tunnel syndrome

The questionnaire was sent to 275 institutions in Austria, the investigators received 118 answered sheets, a result of 43% of feedback. All of these institutions overall performed 866 surgeries on cubital tunnel syndrome. This means an estimated incidence of 0.011% of the Austrian population. Different faculties and surgical specialties were involved in the treatment, the most common one was trauma surgery with 33%. Neurosurgery, for instance, was more rarely involved but in relation to their total number of cases per year cubital tunnel syndrome was the most frequent indication for any operative procedure. For more detail see Figure 4 (Fig. 4).

Endoscopic procedures are part of the treatment-options at only 3 institutions, two of them are plastic surgeons. Among all surgeons the duration of the symptoms was part of their decision, there was an average of 2.75 months until operation.

80% of the surgeons named the clinical examination as their favorite tool for preoperative finding of the diagnosis, but also 80% demanded X-ray of the elbow and electroneurophysiological testing. 40% performed an additional X-ray of the cervical spine. Further examinations, such as CT scan, MRI, or ultrasound were requested in about 10%.

Concerning the non-operative therapy, 40% considered this as useless and if performed, 95% of the patients received physiotherapy, 77% non-steroidal analgetics, or steroid injections (30%).

Regarding the surgical procedures, 70% of the surgeons prefer a single decompression of the nerve, especially in situation of light symptoms, only 4% favor epicondylectomy, resection of the medial epicondylus. At 14 institutions (15%) subcutaneous shift of the nerve was the only option for cubital tunnel syndrome. In complex such as post-traumatic situations or recurrent operation, 62% prefer the subcutaneous shift, 32% the submuscular shift, and 19% epicondylectomy. 

Post-operative immobilisation by a cast-splint is used in 35% for an average of 14 days.

All colleagues performed a follow-up after operation, mostly by clinical examination, 57% added electrophysiological testing.

Major surgical complications were incomplete remission of the symptoms (19%), contraction of scars (13%), and bleeding (12%). The global peri-operative morbidity was assumed by 63%.

## Discussion

Since the 19^th^ century there are documented operations at the ulnar nerve with various tactical options und radicality. In Europe the most common surgery for cubital tunnel syndrome is single decompression of the ulnar nerve. It is less complex than most of the other options, causes less damage at the nerve itself and requires no or only short time immobilization. The latter is accused for adhesions of the nerve to the periostium at the site of the medial epicondyle. Several studies support this opinion [1], [2], [3]. Bultmann et al. published excellent results by endoscopically performed decompression [4].

However, the current treatment for cubital tunnel syndrome is the surgery. There are lots of data concerning the type of operation but there is still no clear evidence to favor anyone of them. The most common surgery is simple nerve decompression, it is safe and leads to good results especially in case of light pre-operative symptoms. In case of recurrence or anatomical reasons for the nerve compression, nerve shift with transposition, most often mostly subcutaneously, and sometimes an epicondylectomy are relevant alternatives.

If there may be an anticipated benefit by shifting the nerve, the best way of nerve transposition, either subcutaneously, intra- or transmuscular, is still in discussion. The investigated institutions favor transposition in cases of recurrence or with additional pathology, but there is no clear evidence for any benefits after nerve transposition yet, even in case of luxation of the nerve in intra-operative testing [5]. 

Epicondylectomy is common in some centers and then performed with comparable results [6], [7], in Austria it is used as an additional option in recurrent compression or pathology in local bone anatomy. 

The data concerning the peri-operative morbidity seem to exceed the expected level, this demands further controlled studies.

## Notes

### Competing interests

The authors declare that they have no competing interests.
